# Reducing Amplified Spontaneous Emission Threshold in CsPbBr_3_ Quantum Dot Films by Controlling TiO_2_ Compact Layer

**DOI:** 10.3390/nano10081605

**Published:** 2020-08-15

**Authors:** Saif M. H. Qaid, Fahhad H. Alharbi, Idriss Bedja, Mohammad Khaja Nazeeruddin, Abdullah S. Aldwayyan

**Affiliations:** 1 Physics and Astronomy Department, College of Science, King Saud University, Riyadh 11451, Saudi Arabia; dwayyan@ksu.edu.sa; 2 Department of Physics, Faculty of Science, Ibb University, Ibb 70270, Yemen; 3 Electrical Engineering Department, King Fahd University of Petroleum and Minerals, Dhahran 31261, Saudi Arabia; fahhad.alharbi@kfupm.edu.sa; 4 K.A. CARE Energy Research & Innovation Center, Dhahran 31261, Saudi Arabia; 5 Cornea Research Chair, Department of Optometry, College of Applied Medical Sciences, King Saud University, Riyadh 11433, Saudi Arabia; bedja@ksu.edu.sa; 6 Group for Molecular Engineering of Functional Materials, Swiss Federal Institute of Technology Lausanne (EPFL), EPFL Valais Wallis, CH-1951 Sion, Switzerland; mdkhaja.nazeeruddin@epfl.ch; 7 King Abdullah Institute for Nanotechnology, King Saud University, Riyadh 11451, Saudi Arabia; 8 K.A. CARE Energy Research and Innovation Center at Riyadh, Riyadh 11451, Saudi Arabia

**Keywords:** amplified spontaneous emission, CsPbBr_3_ laser, CsPbBr_3_ quantum dots, TiO_2_ compact layer

## Abstract

Amplified spontaneous emission (ASE) threshold in ${\mathrm{CsPbBr}}_{3}$ quantum dot films is systematically reduced by introducing high quality ${\mathrm{TiO}}_{2}$ compact layer grown by atomic-layer deposition. Uniform and pinhole-free ${\mathrm{TiO}}_{2}$ films of thickness 10, 20 and 50 nm are used as a substrates for ${\mathrm{CsPbBr}}_{3}$ quantum dot films to enhance amplified spontaneous emission performance. The reduction is attributed indirectly to the improved morphology of ${\mathrm{TiO}}_{2}$ compact layer and subsequently ${\mathrm{CsPbBr}}_{3}$ active layer as grown on better quality substrates. This is quantified by the reduced roughness of the obtained films to less than 5 nm with 50 nm ${\mathrm{TiO}}_{2}$ substrate. Considering the used growth method for the quantum dot film, the improved substrate morphology maintains better the structure of the used quantum dots in the precursor solution. This results in better absorption and hence lower threshold of ASE. Besides that, the improved film quality results further in reducing light scattering and hence additional slight optical enhancement. The work demonstrates a potential venue to reduce the amplified spontaneous emission threshold of quantum dot films and therefore enhanced their optical performance.

## 1. Introduction

Recently, halide perovskites materials attracted a lot of attention; especially for their remarkable deployment in solar cells [1,2,3]. Their general chemical formula is ${\mathrm{ABX}}_{3}$, where A is a monovalent cation, B is a divalent metallic cation, and X is a halogen (or a mix of them). By proper cation and anion substitutions, the physical properties of perovskite material can be modified and tuned [4,5,6,7]. The extraordinary success of perovskites materials in solar cells manifest the possibility of extending that to other optical and optoelectronic applications like as active layers in light-emitting diodes (LEDs) and lasers [8,9,10,11,12].

One of the main practical challenges of halide perovskites is stability. This problem is more pronounced for hybrid perovskites. So, in general, it is expected that full inorganic perovskites are more stable than hybrid ones. For example, it was found that replacing methylammonium monovalent cation by cesium (Cs) results in much better thermal and photostability [13,14,15,16]. Optical-wise, the replacement of iodine (I) with bromide (Br) anion widens the perovskite bandgap to around 2.3 eV. This shall allow the realization of true-green light-emitting devices, which have strong limitations in their present materials and fabrication technologies [15,17].Thus, cesium-based bromide perovskites and in particular ${\mathrm{CsPbBr}}_{3}$ are considered very promising light-emitting materials [18,19,20].

The first observation of the applicability of halide perovskites for stimulated emission as amplified spontaneous emission (ASE) was only achieved in 2014 by Xing et al. [21]. This was followed by developments in many directions. For example, due to the flexible structures, shapes, and sizes of halide perovskites, which can be conveniently manipulated as quantum dots (QDs) and nanowires (NWs), they are exploited to tune lasing properties [5,22,23,24]. Nanostructuring ensures having regular and smooth end faces, which can naturally serve as an active resonant cavity oscillation and optical gain media by themselves. The compact physical sizes from several to tens of microns can confine the generated photons located in the nano-structured cavities, and when the photons satisfy the resonance conditions of microcavities and obtain sufficient gain, the lasing will happen. Thus, perovskite laser is highly promising for integration onto on-chip circuitry because of its small modal volume [25].

Furthermore, due to their remarkably high photoluminescence quantum yield (PLQY), perovskite QDs have attracted the attention as a potential for new generation of gain optical materials for amplified spontaneous emissions (ASEs) and lasers in general [26,27]. In particular, it was found that ${\mathrm{CsPbBr}}_{3}$ exhibits remarkable features and improved stability when compared to ${\mathrm{CsPbI}}_{3}$ and ${\mathrm{CsPbCl}}_{3}$. This fact besides the aforementioned attractive bandgap of 2.3 eV lead most of perovskite QDs light-emitting research to focus on ${\mathrm{CsPbBr}}_{3}$.

In this work, we investigate the effect of improving the morphology of the substrate on ${\mathrm{CsPbBr}}_{3}$ QDs film. This is done by introducing high quality ${\mathrm{TiO}}_{2}$ compact layer grown by atomic-layer deposition (ALD). The compact layer (CL) or buffer layer is an ultrathin high-quality crystalline grown on top of a glass substrate to smooth the surface prior to QDs deposition. In principle, this shall improve the morphology of the subsequently grown ${\mathrm{CsPbBr}}_{3}$ QDs layer. To achieve that, CL must be as smooth as possible, crack free, and having homogeneous optical properties. Thus, as known, ALD can make ultra-thin layers with the desired qualities and properties. We found that CL—indeed–improves the active layer morphology which in turn allows maintaining better the structure of the used quantum dots in the precursor solution. This results in better absorption and hence lower ASE threshold. This is enhanced further with the thickness of CL layer as quantified by the reduced roughness of the obtained films to less than 5 nm with 50 nm ${\mathrm{TiO}}_{2}$ substrate.

We deposited ${\mathrm{TiO}}_{2}$ CL with thicknesses varied from 10 to 50 nm onto the normal glass substrate. The conditions are optimized to have the best possible morphology CLs. After that, ${\mathrm{CsPbBr}}_{3}$ QDs are spin-coated deposited on the prepared substrates. Furthermore, we prepared samples of ${\mathrm{CsPbBr}}_{3}$ QDs without CL. It was found that ASE threshold is reduced by incorporating CL. This improves further with the CL thickness. The threshold –in term of excited carrier density—is reduced from $6.52\times 10^{18}$ cm${}^{-3}$ without CL to $4.59\times 10^{18}$ cm${}^{-3}$ with 50 nm CL. The reduction is attributed indirectly to the improved morphology of ${\mathrm{TiO}}_{2}$ compact layer and subsequently ${\mathrm{CsPbBr}}_{3}$ active layer as grown on better quality substrates. Considering the used growth method for the quantum dot film, the improved substrate morphology maintains better the structure of the used quantum dots in the precursor solution. Beside this, the improved film quality results further in reducing light scattering and hence additional slight optical enhancement. The work demonstrates a potential approach to reduce the amplified spontaneous emission threshold of quantum dot films and hence enhance their performance.

## 2. Materials and Methods

High quality (${\mathrm{CsPbBr}}_{3}$ perovskite QDs (PQDs)/ALD-${\mathrm{TiO}}_{2}$ compact layer/glass) films, which are labeled as [PQDs/c-${\mathrm{TiO}}_{2}$], are used in the present investigation. First, the microscopic glass substrates were washed with soap water under sonicator for 15 min, followed by water only under sonicator for 15 min, then acetone under sonicator for 15 min, and the last one with Isopropyl alcohol (IPA) for 15 min under ultrasonicator. Then, they are left to dry in room temperature. After that, ${\mathrm{TiO}}_{2}$ compact layers werere deposited by ALD (R 200, Picosun, Espoo, Finland) with various layer thicknesses (10 nm, 20 nm, and 50 nm). The details of similar compact layer growth can be found in a previous work [28], where Si(111) was used as substrate.

For ${\mathrm{CsPbBr}}_{3}$ films, high-quality ${\mathrm{CsPbBr}}_{3}$ QDs powder were purchased from Quantum-Solutions Company (Thuwal, Saudi Arabia). Then, the QDs powder was dissolved in a proper amount of hexanes to get the required concentration of 50 mg/mL and left overnight for better dispersion and complete dissolution. Finally, the ${\mathrm{CsPbBr}}_{3}$ QDs thin films were prepared by spin-coating method via 100 μL of QDs solution dropped on the pre-prepared glass/ALD-${\mathrm{TiO}}_{2}$ CL substrates. The drop size was 2 cm${}^{2}$ and the spinning speed was 4000 rpm for 30 s. In order to remove the solvent completely, we just leave it dry under vacuum for an hour.

The structural properties of the grown films were investigated and characterized using High-Resolution Transmission Electron Microscope HRTEM (JEM-2100F, JEOL, Tokyo, Japan) system using an acceleration voltage of 200 kV and X-ray Diffractometer by (Miniflex 600, Rigaku, Tokyo, Japan) system with CuK${}_{\alpha }$ radiation was used in XRD measurements at the scanning angle 2θ was changed between 20${}^{\circ }$ and 50${}^{\circ }$ in steps of 0.01${}^{\circ }$. Atomic Force Microscope (AFM) Characteristics using Multimode V Scan Probe Microscope (Nanoscope V, Veeco, Santa Barbara, CA, USA) in tapping mode was used to examine surface morphologies. Photoluminescence (PL) spectra of the films were carried out with a luminescence spectroscopy system (LS45, Perkin-Elmer, Beaconsfield, UK).

Finally, the incident power-dependent ASE spectra were collected using a Beta-Barium Borate Optical Parametric Generator (OPG) (with a tunable range of 420–2300 nm) (LT-2215 OPG-PC, LOTTIS II, Minsk, Belarus) operated and pumped by a Q-switched Nd:YAG picosecond laser (LS-2151, LOTTIS II, Minsk, Belarus). The pulse duration was about 75 ps, with a repetition rate of 15 Hz. The beam size was focused to around 2mm-radius by a conventional lens. The output signal from the sample was collected through an optical fiber with a collimating lens attached with a spectrograph (QE65 Pro, Ocean Optics, Dunedin, USA). Transient PL measurements were carried out using a laser flash photolysis spectrometer setup (LP920, Edinburgh, Livingston, UK) following the conventional measurements procedure [29,30].

Furthermore, in order to check the prepared samples stabilities, an additional ASE operational stability test has been done. For this regard, the sample was left under continuous laser operation in air for more than an hour at pumping level of energy density beyond the ASE threshold value. PL intensity was automatically measured after each 60 s interval. This can be done by calculating the output intensity by integrating the spectrograph counts across the emission spectrum and then normalizing to create a degradation curve.

## 3. Results

### 3.1. Structural Results

**Figure 1:**
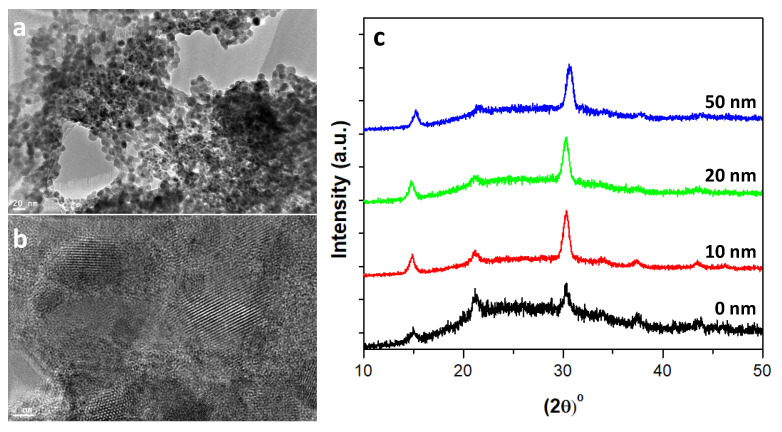
(**a**) TEM Image of the pure ${\mathrm{CsPbBr}}_{3}$ PQDs film, (**b**) High-resolution TEM of the pure ${\mathrm{CsPbBr}}_{3}$ PQDs film, and (**c**) XRD patterns of the parent spectra of the pure ${\mathrm{CsPbBr}}_{3}$ PQDs and ${\mathrm{CsPbBr}}_{3}$ PQDs/(c-TiO_2_) films deposited on glass substrate with various thicknesses of 0, 10, 20, and 50 nm.

Figure 1a,b show low and high resolution transmission electron microscopy (TEM) images of the pure QDs. For TEM characterization, samples were prepared by dilution of 10 μL from ${\mathrm{CsPbBr}}_{3}$ QDs solution with 2 mL of hexane followed by placing several drops of a dilute ${\mathrm{CsPbBr}}_{3}$ QDs solution onto a carbon-coated copper grid. The figures indicate that ${\mathrm{CsPbBr}}_{3}$ QDs were successfully grown. This is further confirmed by the measured XRD patterns shown in Figure 1c. A set of distinct peaks located at approximately $2\theta =15.33^{\circ },21.70^{\circ },30.87^{\circ },34.45^{\circ },37.90^{\circ }\&\;43.93^{\circ }$ that correspond, respectively, to diffractions from (100), (110), (200), (210), (211) and (220) planes of the ${\mathrm{CsPbBr}}_{3}$ perovskite cubic structure [12,19,23,24,31]. Interestingly, there are clearly some differences in the sharpnesses of XRD peaks. These are associated with the QDs sizes, which are deduced from the full width at half maximum (FWHM) of the peaks of $30.87^{\circ }$ using the Scherrer equation. The calculated ${\mathrm{CsPbBr}}_{3}$ QD sizes are listed in Table 1.

**Figure 2:**
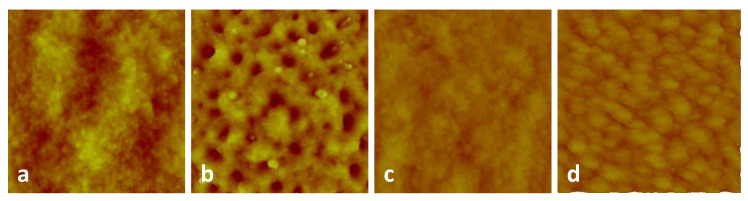
3D- AFM images of 5 × 5 μm scan area of the pure PQDs and PQDs/c-${\mathrm{TiO}}_{2}$ films deposited on glass substrate with various thicknesses (**a**–**d**) for 0, 10, 20, and 50 nm c-${\mathrm{TiO}}_{2}$ thickness respectively.

Then, the morphology of the grown films were characterized using atomic force microscopy (AFM). The 3D- AFM images of 5 × 5 μm scan area are shown in Figure 2 for the different ${\mathrm{TiO}}_{2}$ CL with thicknesses of 0, 10, 20, and 50 nm. The resulted surface roughness obtained by AFM of the grown ${\mathrm{CsPbBr}}_{3}$ PQDs film are shown in Table 2.

### 3.2. Optical Results

**Figure 3:**
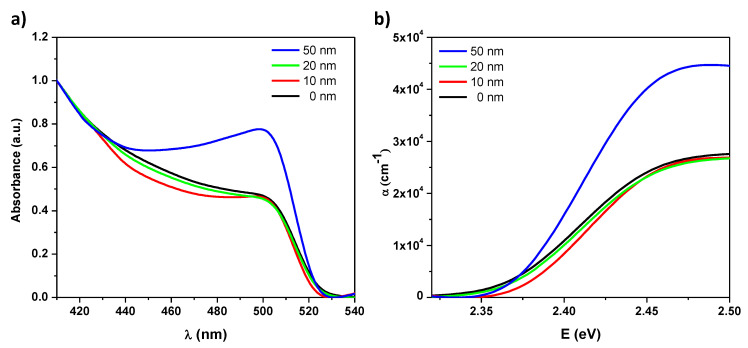
(**a**) Absorbance spectra vs. wavelength and (**b**) absorption coefficient vs. photon energy of the pure PQDs ${\mathrm{CsPbBr}}_{3}$ and ${\mathrm{CsPbBr}}_{3}$ PQDs/c-${\mathrm{TiO}}_{2}$ films of 0, 10, 20, and 50 nm c-${\mathrm{TiO}}_{2}$ thickness respectively.

The optical properties of the grown thin films were then investigated to study the effects of CL thickness on these properties. The absorbance spectra, the PL spectra, and the corresponding transient PL were measured. Figure 3a shows the absorbance spectrum of the studied samples. The absorbance spectrum has a sharp peak at 510 nm. An absorption background of about 0.3 was subtracted from the absorption spectrum in order to remove the wavelength independent component of light scattering [32]. Furthermore, it is clear from Figure 3b that the absorption coefficient increases with CL thickness.

**Figure 4:**
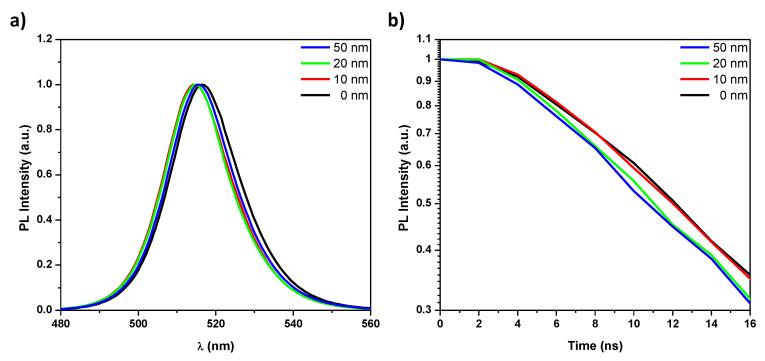
(**a**) PL spectra (normalized) and (**b**) the transient PL decay profiles of the pure PQDs ${\mathrm{CsPbBr}}_{3}$ and ${\mathrm{CsPbBr}}_{3}$ PQDs/c-${\mathrm{TiO}}_{2}$ films of 0, 10, 20, and 50 nm c-${\mathrm{TiO}}_{2}$ thickness respectively.

Figure 4a represents the normalized PL spectra for the grown films. There is a very small variation of the peak position around 515 nm. The small differences are within PL apparatus error margin. Figure 4b shows the PL transient behavior for all the grown films. The faster decay corresponds to intenser emission [33]. The PL decay exhibits a consistent trend as it is faster for thicker CL layers. This has considerable effect in the reduction of the threshold as discussed in the next section.

### 3.3. Amplified Spontaneous Emission Analysis

**Figure 5:**
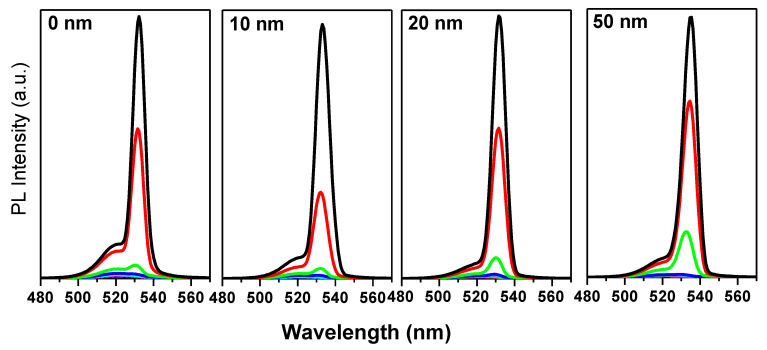
The evolution of PL spectra with pulse energy density of the pump under 410 nm excitation of the pure PQDs ${\mathrm{CsPbBr}}_{3}$ and ${\mathrm{CsPbBr}}_{3}$ PQDs/c-${\mathrm{TiO}}_{2}$ films of 0, 10, 20, and 50 nm c-${\mathrm{TiO}}_{2}$ thickness respectively. (black for 24 μJ/cm${}^{2}$, red for 23.5 μJ/cm${}^{2}$, green for 23 μJ/cm${}^{2}$, blue for 22.5 μJ/cm${}^{2}$, and cyan for 22 μJ/cm${}^{2}$; other lower densities between 19 and 21.5 μJ/cm${}^{2}$ are used in the measurements as well but are not shown here as the differences are insignificant).

ASE was effortlessly observed in all samples; but obviously, with varying efficiencies. Figure 5 shows the PL spectra evolution indicating the presence of two regimes from Spontaneous Emission (SE) to ASE with increasing the pump energy density under 410 nm excitation. Below the threshold energy densities, PL shows relatively broad emission spectra centered around 520 nm for all samples. At and above the threshold, as reported previously [11], this broad emission is split into two bands with the emergence of a narrower peak at around 530 nm, which characterizes the ASE state.

**Figure 6:**
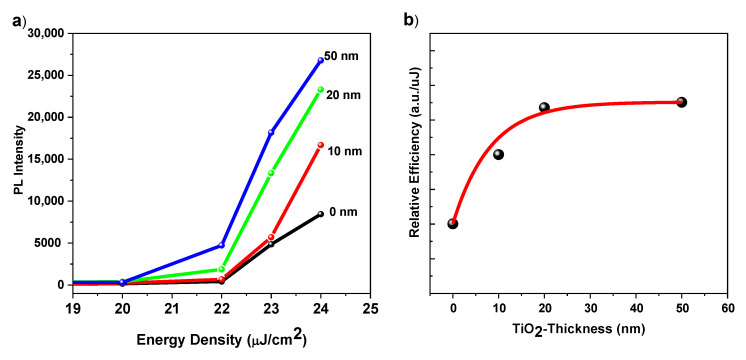
(**a**) The integrated PL intensity vs. pulse energy density and (**b**) the slopes (Differential Quantum Efficiency) of the fitted lines beyond the threshold in intensity-pump energy depending curves as a function of the c-${\mathrm{TiO}}_{2}$ film thickness. In (**b**), the black dots are for the experimental data and the red line is by fitting.

Considering the thickness of the compact layer, it is observed that the intensity evolution of ASE with the pump energy becomes larger as CL thickness increases evidencing the strongly different slope of the PL intensity/energy density curves at various values of CL thickness. The film of 50 nm ${\mathrm{TiO}}_{2}$ CL has shown a higher slope. This difference will be clearer later when discussing the relation between PL versus excited carrier density since the excited carrier density not only depends on the pumping energy but also depends more on the photon absorption value at the pumping energy. This is represented in faster emergence (threshold) of the integrated PL vs. the excited pulse energy density (Figure 6a). The ASE state appeares around 22.17, 21.92, 21.13, and 20.03 μJ/cm${}^{2}$ for ${\mathrm{TiO}}_{2}$ CL thicknesses of 0, 10, 20, and 50 nm respectively. Threshold is estimated by determining the mean value of the minimum pump energy density that allows the observation of the ASE regime measured in 3 different positions on the sample. Also, it is found that the differential quantum efficiency, which can be deduced from the slope of the PL vs. energy density curves, increases with ${\mathrm{TiO}}_{2}$ CL thickness as shown in Figure 6b. This figure shows the slopes of the fitting lines beyond the threshold as a function of c-${\mathrm{TiO}}_{2}$ thickness.

**Figure 7:**
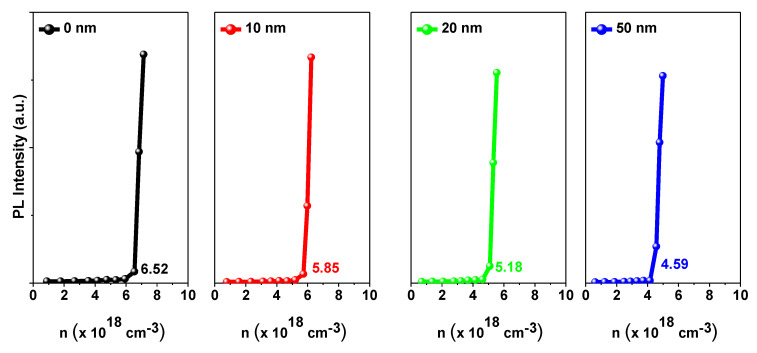
PL versus excited carrier density of the pure PQDs ${\mathrm{CsPbBr}}_{3}$ and ${\mathrm{CsPbBr}}_{3}$ PQDs/c-${\mathrm{TiO}}_{2}$ films for 0, 10, 20, and 50 nm c-${\mathrm{TiO}}_{2}$ thickness respectively.

Finally, the excited carrier density is estimated from the absorbed photon density, which depends on the film thickness and the absorbance (Figure 3). The total number of photons ($N_{exc}$) in the exciting pulse is simply the total pulse energy vs. the energy of individual photon, i.e.:

(1) $$N_{exc}=\frac{E_{pulse}}{\frac{hc}{\lambda_{exc}}}$$

where $E_{pulse}$ is the total pulse energy and $\lambda_{exc}=410$ nm is the exciation wavelength. Then, we need to estimate the number of the absorbed photons, which equals to the number of photons ($N_{exc}$) in the exciting pulse minus the transmitted photons. So [11],

(2) $$N_{abs}=N_{exc}\left(1-e^{-A}\right)$$

where *A* is the absorbance where we assume that non-radiative losses are negligible. From $N_{abs}$, we can estimate the excited carrier density. Figure 7 shows PL intensity vs. the estimated excited carrier density (*n*) for the pure ${\mathrm{CsPbBr}}_{3}$ QDs and ${\mathrm{CsPbBr}}_{3}$ QDs/c-${\mathrm{TiO}}_{2}$ films of 0, 10, 20, and 50 nm c-${\mathrm{TiO}}_{2}$ thickness respectively. This quantifies ASE threshold. Clearly, ASE threshold decreases with ${\mathrm{TiO}}_{2}$ CL thickness.

**Figure 8:**
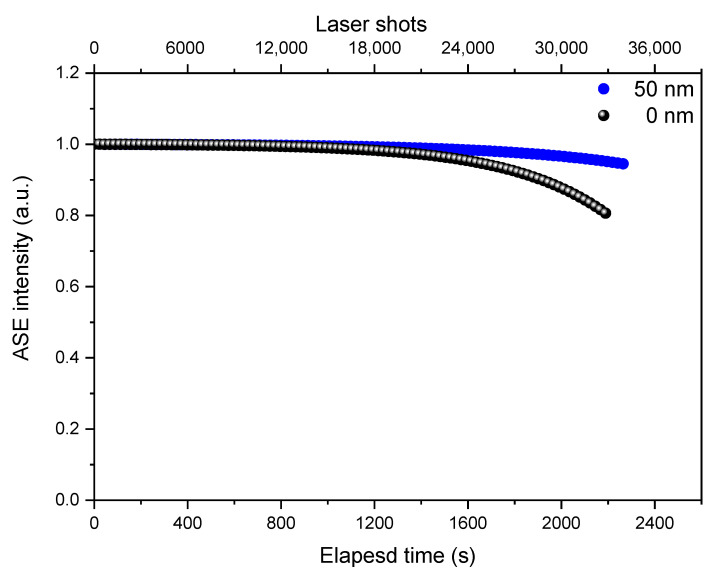
ASE operational stability behavior for ${\mathrm{TiO}}_{2}$ CL thicknesses of 0 and 50 nm samples.

Finally, ASE operational stability has been investigated by measuring the PL spectra above the ASE threshold during continuous pumping in air. Considering that in real laser applications a device is operated at an excitation density that allows stable light amplification. Remembering that, the ASE threshold quantifies the minimum excitation density necessary to have light amplification and the ASE operational lifetime, quantified as the number of pump laser pulses necessary to have an ASE intensity reduction of a factor e (1/e lifetime). So, we measured ASE intensity stability under continuous laser pumping at an excitation energy level 2 times larger than ASE threshold value for all the samples. In particular, the excitation density was set at 44.34, 43.84, 42.26, and 40.06 μJ/cm${}^{2}$ for ${\mathrm{TiO}}_{2}$ CL thicknesses of 0, 10, 20, and 50 nm respectively. Figure 8, showed a nice stable ASE intensity up to about 23250 laser pulses for ${\mathrm{TiO}}_{2}$ CL thicknesses of 0 and 50 nm. A progressive intensity decrease at longer times for ${\mathrm{TiO}}_{2}$ CL thicknesses of 0 nm could be noticed. From the extrapolation of the experimental data, the film of 50 nm c-${\mathrm{TiO}}_{2}$ thickness shows an excellent ASE intensity stability, with 1.2 times higher than the film of 0 nm c-${\mathrm{TiO}}_{2}$ thickness. In particular, after 33,000 pulses ASE intensity of the film of 50 nm c-${\mathrm{TiO}}_{2}$ thickness is still preserving 95% of the initial value, while it became 80% of the initial value in case of 0 nm c-${\mathrm{TiO}}_{2}$ thickness. So, we should thus expect a long ASE operational lifetime (1/e) in non-encapsulated films operating in air demonstrates a remarkable photo-stability of the samples deposited on c-${\mathrm{TiO}}_{2}$.

## 4. Discussion

At the beginning, the XRD patterns (Figure 1c) and TEM images (Figure 1a,b) confirm that the grown of ${\mathrm{CsPbBr}}_{3}$ QDs films are of high phase purity. Yet, the estimated final QDs sizes (Table 1) from XRD peak around $2\theta =30.87^{\circ }$ indicate that they are smaller for thicker ${\mathrm{TiO}}_{2}$ CL. Furthermore, the morphology analysis illustrates that the film morphology improves with ${\mathrm{TiO}}_{2}$ CL thickness. This is quantified by the reduced roughness (Table 2). Considering the used growth method for the quantum dot film, the improved substrate morphology maintains better the structure of the used quantum dots in the precursor solution (smaller QDs), i.e., less distortion and more aggregation. This behavior was occasionally observed for QDs films [34,35,36]. However, a further detailed investigation is needed for better quantification and interpretation. This is beyond the scope of this study and should be done in a future separate work.

This preservation of the original structure of ${\mathrm{CsPbBr}}_{3}$ QDs results in more suitable optical properties for ASE as the absorbance increases with ${\mathrm{TiO}}_{2}$ CL thickness. This can be attributed to the increased QDs density due to the aggregation of smaller QDs. It is clear from Figure 3b that the absorption coefficient increases with CL thickness, which resulted—in turn—in faster transient PL decay as shown in Figure 4b. As aforementioned, the faster decay corresponds to an intenser emission [33]. Distinctly, the PL decay exhibits a consistent trend as it is faster for thicker CL layers.

Furthermore, the improved film morphology (i.e., smaller roughness) shall reduce incident light scattering. Thus, it is expected that more pump energy enters to films with lower roughness (i.e., thicker CT) and hence improved overall absorption as certainly observed (Figure 3). As aforementioned, this resulted further in faster transient PL decay as shown in Figure 4b and hence lower ASE threshold.

This improved absorption and the subsequent faster emission are the essence of the observed reduction in the ASE threshold. As shown in Figure 7, the estimated threshold excited densities are $6.52\times 10^{18}$, $5.85\times 10^{18}$, $5.18\times 10^{18}$, and $4.59\times 10^{18}$ for ${\mathrm{TiO}}_{2}$ CL thicknesses of 0, 10, 20, and 50 nm respectively. The estimated threshold excited densities have been estimated by determining the mean value of the excitation density, measured at 3 different positions on the sample. This is corresponds to 10.28%, 20.55%, and 29.60% reductions in ASE threshold for 10, 20, and 50 nm CL thickness. So, the presented approach demonstrated a very high potential to reduce ASE threshold of quantum dot films and hence to enhance their optical performance.

## Figures and Tables

**Table 1:** The calculated ${\mathrm{CsPbBr}}_{3}$ QD sizes from XRD patterns using the Scherrer equation.

c-$TiO_{2}$ Thickness (in nm)	FWHM (in degree)	QD Diameter (in nm)
0	0.433	19.00
10	0.606	13.57
20	0.635	12.96
50	0.693	11.89

**Table 2:** The surrface roughness of the grown ${\mathrm{CsPbBr}}_{3}$ QD films vs. CL thickness.

c-$TiO_{2}$ Thickness (in nm)	Surface Roughness (in nm)
0	14.0
10	21.5
20	16.7
50	5.8
